# Application of metagenomic next-generation sequencing in the diagnosis of severe pneumonia caused by *Chlamydia psittaci*

**DOI:** 10.1186/s12890-021-01673-6

**Published:** 2021-09-23

**Authors:** Huan-huan Wu, Lan-fang Feng, Shuang-yan Fang

**Affiliations:** Department of Respiratory Medicine, Dongyang Hospital Affiliated to Wenzhou Medical University, Dongyang, 322100 Zhejiang Province China

**Keywords:** *Chlamydia psittaci*, Severe pneumonia, mNGS, Tetracyclines

## Abstract

**Purpose:**

Psittacosis is a zoonotic infectious disease caused by the transmission of the bacterium *Chlamydia psittaci* (*C. psittaci*) from birds to humans. Infections in humans mainly present as community-acquired pneumonia (CAP). However, most cases are treated without diagnostic testing, and the importance of *Chlamydia psittaci* infection as a cause of CAP is therefore unclear. Diagnostic tools, including culture, serologic test, and PCR-based methods, are available but prone to false negative results. Metagenomic next-generation sequencing (mNGS) has been increasingly used in the diagnosis of infectious diseases, particularly when conventional diagnostic approaches have limitation.

Detection of nucleic acid sequence of *C. psittaci* in respiratory tract samples by metagenomic next-generation sequencing (mNGS) is effective for early diagnosis of severe *C. psittaci* pneumonia. Timely treatment based on tetracycline can reduce unnecessary use of antibiotics and improve prognosis of patients with severe *C. psittaci* pneumonia.

**Methods:**

Clinical data of thirteen patients with severe *C. psittaci* pneumonia diagnosed by mNGS were collected. Clinical manifestations, treatment and prognosis of patients were summarized.

**Results:**

The typical symptoms of pneumonia caused by *C. psittaci* include fever, headache, myalgia, cough, and dyspnea. In the current study, all patients met the criteria for severe *C. psittaci* pneumonia and received mechanical ventilation, including noninvasive mechanical ventilation (five/thirteen) and invasive mechanical ventilation (eight/thirteen). The findings showed that patients with *C. psittaci* pneumonia presented with normal or slightly increased leucocytes and procalcitonin, and high C-reactive protein levels. Computed tomography manifestations included consolidation of lung parenchyma, with air bronchogram and pleural effusion in some patients. mNGS analysis results were obtained within 48–72 h. Eleven patients fully recovered after targeted treatment, however, two patients died from secondary multidrug-resistant *Pseudomonas aeruginosa* infection.

**Conclusions:**

The findings of the current study show that mNGS is effective in diagnosis of *C. psittaci* pneumonia, and has significant diagnosis value in patients with severe infection. Patients responds well to the timely use of appropriate antibiotics.

## Introduction

*Chlamydia psittaci* is a gram-negative, obligate intracellular bacterium. Infection by *C. psittaci* is transmitted to human beings mainly through contact or inhalation of aerosol, feces, or feather dust from nasal secretions of infected birds [1–3]. The primary host of *C. psittaci* is various birds including parrots and pigeons. In addition, mammals and poultry are potential hosts of the bacterium and can readily transmit it to people [1–3]. *C. psittaci* infection in humans is mainly an occupational infection. Moreover, person-to-person transmission has been reported, however, it is extremely rare [4–7].

*C. psittaci* mainly causes respiratory and digestive tract diseases in birds. In humans, it causes respiratory tract infection and bacteremia. *C. psittaci* pneumonia is prevalent among adults and rare in children, accounting for approximately 1% of all community-acquired pneumonia [8]. The disease has an incubation period of five to fourteen days [9]. It mainly invades the lungs, liver, spleen, meninges, and central nervous system. The main symptoms of pneumonia caused by *C. psittaci* include fever (body temperatures above 38.5 ℃), headache, myalgia, cough, and dyspnea. *C. psittaci* can be manifested as asymptomatic to mild infection, resulting in systemic multiple organ dysfunction, severe pneumonia, and may result in death [10–13].

Diagnostic methods currently used for *C. psittaci* include pathogen culture, serological detection, and molecular biology techniques including polymerase chain reaction (PCR). Diagnosis of *C. psittaci* pneumonia is challenging in early stages owing to its atypical clinical manifestations. The low sensitivity and complex procedure of *Chlamydia psittaci* culture causes it hardly routinely performed in most diagnostic laboratories. Other laboratory testing included serological assay and polymerase chain reaction (PCR) based methods, but both have questionable sensitivity and specificity. Metagenomic next-generation sequencing technology (mNGS) can detect various pathogenic microorganisms without bias through sequencing and analysis of microorganisms as well as host nuclear acid in clinical samples. Rapid screening of pathogens facilitates timely identification of pathogens to initiate targeted antibiotic treatment. Therefore, mNGS has been gradually applied in clinical practice in recent years [14, 15]. A total of 13 cases of severe pneumonia caused by *C. psittaci* diagnosed using mNGS method in Dongyang Hospital Affiliated to Wenzhou Medical University are presented in the current study.

## Patients and methods

### Study design

Clinical data of thirteen patients with severe pneumonia caused by *C. psittaci* admitted to Dongyang Hospital Affiliated to Wenzhou Medical University (a tertiary hospital in Zhejiang, China) between January 2019 and January 2021 were retrospectively analyzed in the current study. All the cases were diagnosed by mNGS combined with clinical manifestations and other laboratory diagnostic methods. Clinical data of each patient were obtained from the electronic medical record system. The current study was approved by the hospital ethics committee (Approval No.2021-YX-035), and all data were anonymized before analysis.

### mNGS analysis

(1) sample processing and DNA extraction: Samples were collected in strict accordance with clinical practice. All samples were bronchoalveolar lavage fluid. DNA was extracted using the Tianamp micro DNA Kit (DP316, Tiangen Biotech, Beijing, China). (2) Library construction and sequencing: Agilent 2100 Bioanalyzer was used to analyze the length of inserted fragments in the library. Qubit dsDNA HS assay Kit (Thermo Fisher Scientific Inc.) was used to determine the concentration of DNA in the library. DNA nano ball (DNB) nanospheres were produced from the library after cyclization. Further, the prepared DNB nanospheres were loaded into the sequencing chip and sequenced using BGISEQ-500 system. (3) Statistical analysis: Sequencing data were retrieved from the machine and low-quality data, and data with length less than 35 bp were removed to obtain high-quality data. Human reference genome sequence data were retrieved by alignment in BWA webserver (BWA: http://bio-bwa.sourceforge.net/). Low complexity sequences were excluded and the remaining data were compared with the data in the special microbial database. Data were then classified and arranged based on bacteria, fungi, viruses, and parasites.

### Diagnostic criteria

In the current study, severe pneumonia cases were diagnosed as caused by *C. psittaci* if they met the following conditions: (1) The diagnostic criteria of severe pneumonia [16]; (2) detection of the specific DNA fragment of *C. psittaci* through mNGS; (3) no other pathogens were observed in other specimens including blood, sputum, and bronchoalveolar lavage fluid.

## Results

### Patient characteristics

The thirteen patients included in the current study comprised seven males and six females. The median age of patients was 65 (range 33–78) years (Table 1). All patients had an acute onset of pneumonia. Fever was the most common symptom among patients, and the peak temperature was 39–40 ℃. Most patients presented with cough without apparent expectoration. All patients developed progressive dyspnea with disease progression. Patients included in the study did not receive hormones or immunosuppressants. All thirteen patients presented with an episode of respiratory failure during hospitalization and required mechanical ventilation (Table 1).

**Table 1:** Clinical characteristics of the severe psittacosis pneumonia cases

Characteristics	Patients, n (%)	Median value, (range)
*Demographics*		
Male/female	7/6	
Age, median (range, years)		65 (33–78)
History of contact with avian or poultry	5/13 (38.46)	
Underlying disease	5/13 (38.46)	
*Clinical manifestations*		
Fever > 38.5 °C	13/13 (100.00)	
Cough, dyspnea	13/13 (100.00)	
Headache	6/13 (46.15)	
Myalgia	10/13 (76.92)	
Septic shock	9/13 (69.23)	
Invasive ventilator support	5/13 (38.46)	
Days from illness to respiratory failure		8 (2–10)
*Laboratory testing*		
Elevated WBC (normal 4–10, × 10^9^/L)	2/13 (15.38)	7.50 (3.20–18.28)
Elevated percentage of neutrophils (normal 45–75%)	13/13 (100.0)	0.90 (0.63–0.97)
Elevated CRP (normal 0–8mg/L)	13/13 (100.0)	172.87(45.99–270.00)
Increased PCT (normal 0–0.5 ng/ml)	9/13 (69.23)	3.96 (0.06–25.78)
Imaging		
Lesion began in lower lobe of lung	8/13 (61.54)	
Lesion began in upper lobe of lung	5/13(38.46)	
Consolidation with air bronchograms	13/13 (100.00)	
Complete CT recovery in survivors	11/11 (100.00)	
*Oxygen therapy method *		
noninvasive ventilator	8/13(61.5)	
invasive ventilator	5/13(38.46)	

*CRP* C-reactive protein, *CT* computed tomography, *PCT* procalcitonin, *WBC* white blood cell

Positive findings of physical examination included increased tactile tremor in the affected lung and a little moist rale in auscultation, which was not consistent with severe clinical manifestations.

### Technical investigations

Two cases presented with elevated white blood cell (WBC) count, ten cases presented with normal WBC count, whereas one case presented with decreased WBC count during admission. Average WBC count of the thirteen patients was 7.50 × 10^9^/L (normal 4.00–10.00 × 10^9^/L). The mean percentage of neutrophils was 90%; with an average C-reactive protein (CRP) level of 172.87 mg/L (normal < 8 mg/L), and an average procalcitonin level of 3.96ng/ml (normal < 0.5ng/ml).

**Fig. 1:**
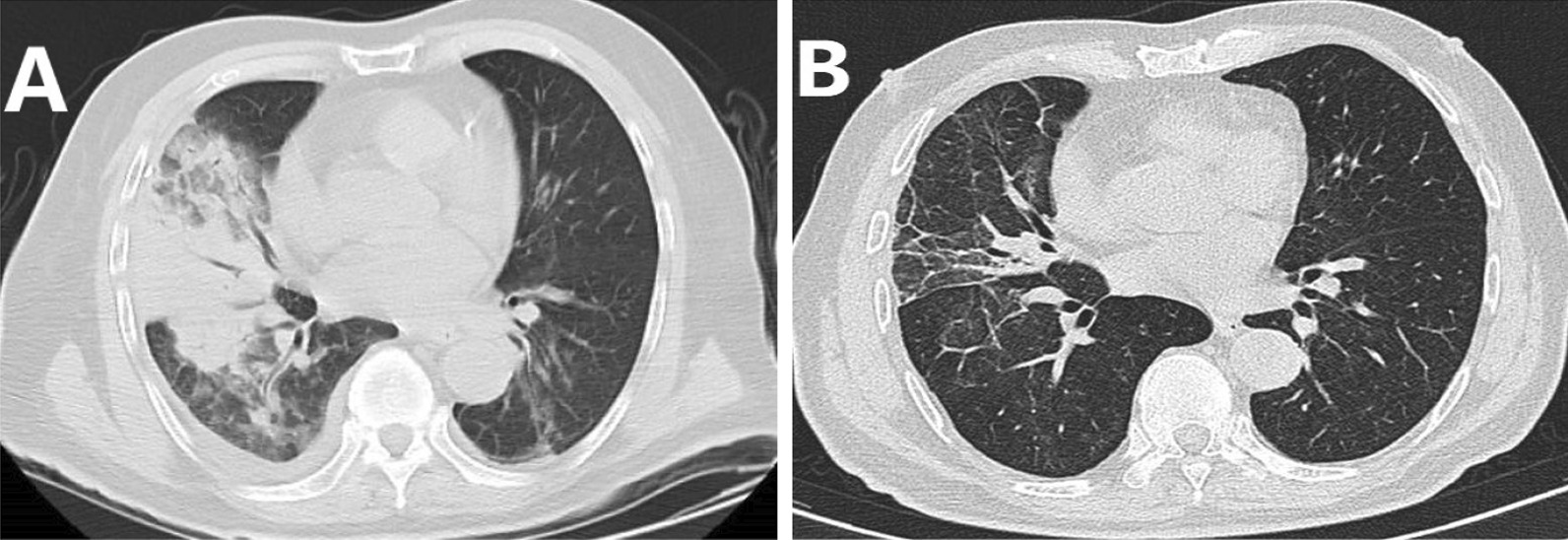
Chest computed tomography (CT) scans of a 78-year-old man with severe *psittacosis* pneumonia. The initial CT scan (11 days after onset) shows air-space consolidation with inflammatory exudation in the superior lobe of the right lung and pleural effusion (**A**). On follow-up, the consolidation was completely absorbed (19 days after onset) (**B**)

**Fig. 2:**
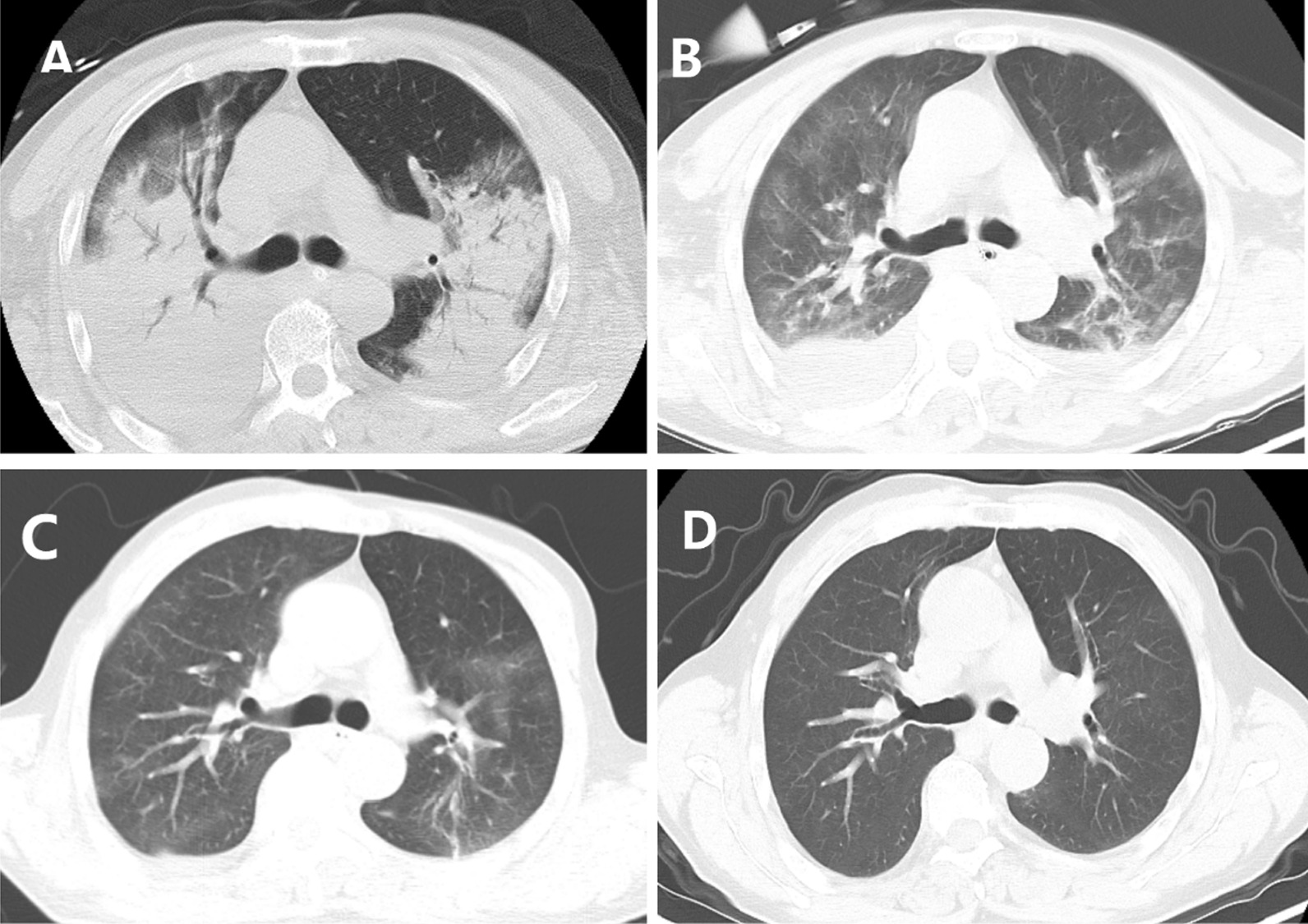
Chest computed tomography (CT) scans of a 69-year-old man with severe *psittacosis* pneumonia. The initial CT scan (6 days after onset) shows diffuse consolidation of both lungs with bronchial inflation sign (**A**). CT scan (14 days after the onset) shows that the consolidation gradually decreased following targeted treatment, however it showed bilateral pleural effusion (**B**). On follow-up (21 days after the onset), the consolidation and pleural effusion gradually decreased following treatment (**C**), and it completely disappeared at 66 days after onset (**D**)

Main computer tomography (CT) manifestations included consolidation with bronchial inflation sign, ground glass exudation, or pleural effusion (Figs. 1 and 2). Pleural effusion was observed in two patients. Two patients died from secondary multidrug-resistant infection after treatment with targeted drugs. The other eleven patients were completely recovered.

### Treatment

All the patients had visited the local community hospital at the onset of the disease and received empirical treatment. However, their condition gradually deteriorated, leading to progression of acute respiratory distress syndrome. Patients were subjected to bronchoscopy examination for the first time at our hospital and underwent bronchoalveolar lavage fluid (BALF) mNGS test.

mNGS analysis takes 48–72 h thus the patients received empirical antibiotic treatment after admission, including β-lactam/β-lactamase inhibitor combinations or quinolones following the guidelines for diagnosis and treatment of community-acquired pneumonia. After confirmation that the disease was caused by *C. psittaci*, antibiotic therapy was changed to tetracycline [9]. In addition, three patients with severe illness were treated with supplementary carbapenems, linezolid, or tigecycline. Clinical studies recommend that the course of tetracycline treatment should be at least 2 weeks [9].

### Outcomes

The body temperature of patients decreased to normal levels within three days after tetracycline treatment. Moreover, tetracycline treatment gradually improved the respiratory function of patients. Two patients died from secondary multidrug-resistant *Pseudomonas aeruginosa* infection which caused a progressive decrease in blood pressure. Notably, hypoxemia could not be corrected even with extracorporeal membrane oxygenation (ECMO) resulting in death. The other patients showed complete recovery.

## Discussion

Psittacine commonly known as parrots are the main hosts of *C. psittaci*. In addition, birds and commercial poultry are potential hosts of *C. psittaci* and can transmit the disease to humans. Contact with infected birds, feathers, tissues, or corpses of birds, and cleaning of contaminated cages predisposes individuals to *C. psittaci* infection [9, 17, 18]. *C. psittaci* infection can be caused by short-term contact with birds or their excreta, and activities that do not involve direct contact with excreta including mowing or pruning [19, 20].

*C. psittaci* pneumonia accounts for appoximately 1% of all community-acquired pneumonia [8]. The mortality rate of *C. psittaci* pneumonia was 15–20% before the era of antibiotics [21]. Notably, the mortality rate has significantly decreased with the advent and development of targeted antibiotics.

Diagnosis of *C. psittaci* pneumonia is challenging owing to its atypical clinical manifestations. For instance, a few patients may not have a history of bird contact. Patients are commonly treated for common community-acquired pneumonia when clinicians do not carefully collect medical history or if the patients report lack of contact history. Furthermore, detection of *C. psittaci* is not often included in the scope of routine detection, resulting in misdiagnosis. In addition, several patients visit the hospital at an early stage of the disease and are mainly treated with empirical antibiotics owing to the hierarchical diagnosis and treatment system. Moreover, the disease has a self-limiting nature. These factors potentially lead to underestimation of the incidence rate of *Chlamydia* pneumonia.

Main clinical manifestations of *C. psittaci* pneumonia include fever, chills, headache, general discomfort, and myalgia. In addition, patients may present with a dry cough and progressive dyspnea or chest tightness. Moreover, a pulse temperature dissociation (rarely without increased pulse rate), splenomegaly, or nonspecific rash observed in patients. Furthermore, *C. psittaci* is associated with several extrapulmonary manifestations, including endocarditis, myocarditis, hepatitis, arthritis, keratoconjunctivitis, encephalitis, and ocular adnexal lymphoma [22–24]. Pulmonary auscultation is ineffective and often inconsistent with severe clinical manifestations. Laboratory analysis shows that the white blood cell count in patients with *C. psittaci* pneumonia is significantly lower compared with other pneumonia types [25]. In the current study, the average white blood cell count was normal, however, the CRP levels were significantly higher compared with the normal value. This finding is consistent with findings from other studies. The major imaging manifestations include consolidation of the air cavity, ground glass shadow, grid shadow, small patches and nodules, pleural effusion, and lobular distribution, with manifestation highly common in the lower lobes of lungs [24, 26]. Multiple image forms may coexist, indicating that the disease gradually develops from bronchioles to surrounding lobules and overlaps with each other. In the present study, five cases showed manifestation of the disease in the upper lobe, whereas eight cases presented distribution in the lower lobe.

Positive diagnosis of *C. psittaci* requires at least one of the following tests: (1) Isolation of *C. psittaci* from respiratory tract specimens; (2) Antibody titer of double serum is 4-fold higher or more compared with the normal value as shown by complement binding test (CFT) or micro immunofluorescence (MIF); relative titer of IgM of *C. psittaci* detected by MIF method is more than 1:16. Notably, only a few microbiological laboratories have facilities for conducting these tests. Microbiological culture is time-consuming, and requires a P3 containment laboratory [9]. Polymerase chain reaction (PCR) is used for quick identification of acute and asymptomatic patients and helps identify the source of disease through genotyping. Notably, sensitivity of PCR is higher in the acute stage of the disease, whereas sensitivity rapidly decreases with progression of the disease. In addition, not all laboratories have facilities for performing PCR analysis. The serological detection method is limited by cross-reaction with other *Chlamydia* strains. It is used for detection of the bacteria in serum samples of patients in acute and convalescent stages. Therefore, serological detection method is only suitable for retrospective diagnosis and can be used for epidemiological investigation [27]. Notably, each detection method has its limitations, including low sensitivity, cross-reactivity with related species and inefficiency in collecting the optimal clinical specimens at the optimal time intervals for subsequent tests. Therefore, detection is often performed through combination of various laboratory detection methods. Moreover, there is no a “gold standard” method for diagnosis [28] resulting in low diagnosis rates. *C. psittaci* infection among elderly patients is more severe compared with infection with pneumonia caused by other *Chlamydia* strains, and the course of disease spans several weeks. Studies report that *C. psittaci* caused outbreaks in several places in Europe such as Belgium and Netherlands in the past [29, 30], thus the impact of the disease on human beings cannot be underestimated. Moreover, there is an urgent need for convenient, rapid, and accurate detection methods to ensure timely and effective treatment.

All patients in the current study were diagnosed through mNGS after presenting with no improvement after antibiotic treatment. Notably, mNGS accurately detects pathogens in patients with unexplained lower respiratory tract infection and the results can be obtained within 48–72 h. The number of pathogen sequences detected by mNGS in BALF was more compared with that in sputum. Notably, there was no interference with oral colonization bacteria implying that it used as the preferred sample. mNGS method is characterized by timely and accurate detection of pathogens. Therefore, mNGS helps in facilitating timely and targeted treatment in clinical setups. Moreover, it effectively shortens the diagnosis time of the disease, mainly for patients with severe pneumonia caused by *C. psittaci*, thus presenting a significant diagnostic value.

Tetracycline is the first-line treatment option for *C. psittaci* pneumonia. However, macrolides can be used if tetracyclines are contraindicated (in children, pregnant women, or allergies). In some cases, quinolones are effective, however, they are less effective compared with tetracycline and macrolides [31]. In the current study, four cases were initially treated with quinolones. However, the therapy was not effective thus the treatment was changed to tetracycline drugs, and finally, the patients recovered and were discharged. Targeted antibiotic treatment decreases *C. psittaci* pneumonia mortality to less than 1%. The mortality rate can reach 10–20% if inappropriate treatment is used [29]. Symptoms are rapidly alleviated with one-two days after administration of targeted antibiotics, and the treatment should be continued for at least fourteen days [29]. Hospitalized patients with severe community acquired pneumonia (CAP) may require intravenous administration with a combination of tetracycline and quinolones [29].

The main limitation of this study is that it was retrospective in nature and included only thirteen cases presenting with severe psittacosis pneumonia. The relatively small sample size is insufficient to explore all relevant features of psittacosis pneumonia. A prospective study of severe psittacosis pneumonia is ongoing, to further explored the feature of the disease and to explore effective therapies.

## Conclusions

Clinicians should be aware of the possibility of *C. psittaci* pneumonia among those with highly suspected clinical symptoms since not all patients with *C. psittaci* pneumonia can recall history of bird contact. All cases in the present study were patients diagnosed with severe pneumonia. Two patients succumbed even after targeted treatment. The findings of the study show that *C. psittaci* pneumonia is associated with adverse outcomes. Metagenomic next-generation sequencing technology (mNGS) allows early detection thus improving prognosis of patients with severe pneumonia associated with an unknown pathogen, thus it is recommended for accurate detection of pathogens.

## Data Availability

All the data supporting the findings of the study is has been reported in the manuscript.

## Abbreviations

BALF: Bronchoalveolar lavage fluid
CRP: C-reactive protein
CT: Computed tomography
mNGS: Metagenomic next-generation sequencing
PCR: Polymerase chain reaction
PCT: Procalcitonin
WBC: White blood cell
CRP: C-reactive protein
ECMO: Extracorporeal membrane oxygenation
MIF: Micro immunofluorescence
CAP: Community acquired pneumonia

## References

[1] Kalmar ID, Dicxk V, Dossche L (2014). Zoonotic infection with Chlamydia psittaci at an avian refuge centre. Vet J.

[2] Magnino S, Haag-Wackernagel D, Geigenfeind I (2009). Chlamydial infections in feral pigeons in Europe: review of data and focus on public health implications. Vet Microbiol.

[3] Haag-Wackernagel D, Moch H (2004). Health hazards posed by feral pigeons. J Infect.

[4] Hughes C, Maharg P, Rosario P (1997). Possible nosocomial transmission of psittacosis. Infect Control Hosp Epidemiol.

[5] Wallensten A, Fredlund H, Runehagen A. Multiple human-to-human transmission from a severe case of psittacosis, Sweden, January-February 2013. Euro Surveill. 2014;19(42):20937. 10.2807/1560-7917.es2014.19.42.20937 25358043

[6] Ito I, Ishida T, Mishima M (2002). Familial cases of psittacosis: possible person-to person transmission. Intern Med.

[7] McGuigan CC, McIntyre PG, Templeton K (2012). Psittacosis outbreak in Tayside, Scotland, December 2011 to February 2012. Euro Surveill.

[8] Hogerwerf L, DE Gier B, Baan B (2017). Chlamydia psittaci (psittacosis) as a cause of community-acquired pneumonia: a systematic review and meta-analysis. Epidemiol Infect.

[9] Balsamo G, Maxted AM, Midla JW (2017). Compendium of measures to control chlamydia psittaci infection among humans (Psittacosis) and pet birds (Avian Chlamydiosis), 2017. J Avian Med Surg.

[10] Heddema ER, van Hannen EJ, Dium B (2006). An outbreak of psittacosis due to Chlamydophila psittaci genotype A in a veterinary teaching hospital. J Med Microbiol.

[11] Stewardson AJ, Grayson ML, Psittacosis (2010). Infect Dis Clin North Am.

[12] Kovácová E, Majtán J, Botek R, et al. A fatal case of psittacosis in Slovakia, January 2006. Euro Surveill. 2007 Aug 2;12(8):E070802.1. 10.2807/esw.12.31.03244-en 17880885

[13] Yilmazlar A, Ozcan B, Kaplan N (2000). Adult respiratory distress syndrome caused by psittacosis. Turk J Med Sci.

[14] Schlaberg R, Chiu CY, Miller S (2017). Validation of metagenomic next-generation sequencing tests for universal pathogen detection. Arch Pathol Lab Med.

[15] Langelier C, Kalantar KL, Moazed F (2018). Integrating host response and unbiased microbe detection for lower respiratory tract infection diagnosis in critically ill adults. Proc Natl Acad Sci USA.

[16] Chen X, Cao K, Wei Y (2020). Metagenomic next-generation sequencing in the diagnosis of severe pneumonias caused by Chlamydia psittaci. Infection..

[17] Fraeyman A, Boel A, Van Vaerenbergh K (2010). Atypical pneumonia due to Chlamydophila psittaci: 3 case reports and review of literature. Acta Clin Belg.

[18] Lagae S, Kalmar I, Laroucau K (2014). Emerging Chlamydia psittaci infections in chickens and examination of transmission to humans. J Med Microbiol.

[19] Williams J, Tallis G, Dalton C (1998). Community outbreak of psittacosis in a rural Australian town. Lancet.

[20] Telfer BL, Moberley SA, Hort KP (2005). Probable psittacosis outbreak linked to wild birds. Emerg Infect Dis.

[21] Dunnahoo GL, Hampton BC (1945). Psittacosis: occurrence in the United States and report of 97 % mortality in a shipment of birds while under quarantine. Public Health Rep.

[22] Schlossberg D, Bennett JE, Dolin R, Blaser MJ (2015). Psittacosis (due to Chlamydia psittaci). Mandell, Douglas, and Bennett’s Principles and Practice of Infectious Diseases.

[23] Beeckman DS, Vanrompay DC (2009). Zoonotic Chlamydophila psittaci infections from a clinical perspective. Clin Microbiol Infect.

[24] Zucca F, Bertoni F (2006). Chlamydia or not Chlamydia, that is the question: which is the microorganism associated with MALT lymphomas of the ocular adnexa?. J Natl Cancer Inst.

[25] Vande Weygaerde Y, Versteele C, Thijs E (2018). An unusual presentation of a case of human psittacosis. Respir Med Case Rep.

[26] Longbottom D, Coulter LJ (2003). Animal chlamydioses and zoonotic implications. J Comp Pathol.

[27] Spoorenberg SM, Bos WJ, van Hannen EJ (2016). Chlamydia psittaci: a relevant cause of community-acquired pneumonia in two Dutch hospitals. Neth J Med..

[28] Nieuwenhuizen AA, Dijkstra F, Notermans DW, et al. Laboratory methods for case finding in human psittacosis outbreaks: a systematic review. BMC Infect Dis. 2018;18(1):442. 10.1186/s12879-018-3317-0 PMC6118005 30165831

[29] Rybarczyk J, Versteele C, Lernout T (2020). Human psittacosis: a review with emphasis on surveillance in Belgium. Acta Clin Belg.

[30] de Gier B, Hogerwerf L, Dijkstra F (2018). Disease burden of psittacosis in the Netherlands. Epidemiol Infect.

[31] DE Boeck C, Dehollogne C, Dumont A (2016). Managing a cluster outbreak of psittacosis in Belgium linked to a pet shop visit in The Netherlands. Epidemiol Infect..

